# Supplementary material for: Robust RNA-based in situ mutation detection delineates colorectal cancer subclonal evolution
Source: Nat Commun. 2017 Dec 8;8:1998. doi: 10.1038/s41467-017-02295-5 (PMC5722928; doi:10.1038/s41467-017-02295-5)
Supplement: Supplementary file 1 — Supplementary Information [file 41467_2017_2295_MOESM1_ESM.pdf]

Supplementary Figure 1. Baker *et al*
Validation of BaseScope probes in cell lines

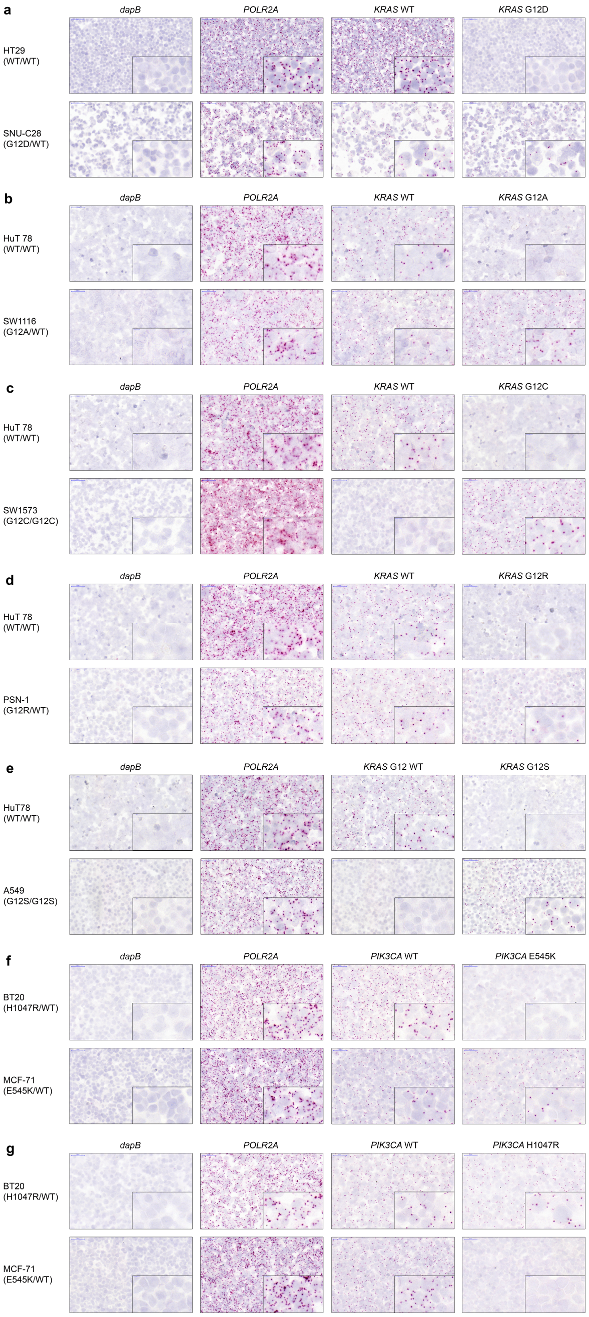

Supplementary Figure 1 – Validation of BaseScope probes in cell lines
Representative images of the validation of the *KRAS* G12D probe set (**a**), *KRAS* G12A probe set (**b**), *KRAS* G12C probe set (**c**), *KRAS* G12R probe set (**d**), *KRAS* G12S probe set (**e**), *PIK3CA* E545K probe set (**f**) and *PIK3CA* H1047R probe set (**g**). In all cases a wild-type cell line and a heterozygous/homozygous mutant cell line are shown, with a negative control probe (*dapB*), a positive control probe (*POLR2A*), the wild-type probe and the mutant probe. Probe binding is visualized as punctate red dots. Scale bars represent 50 micron and 10 micron (inset).

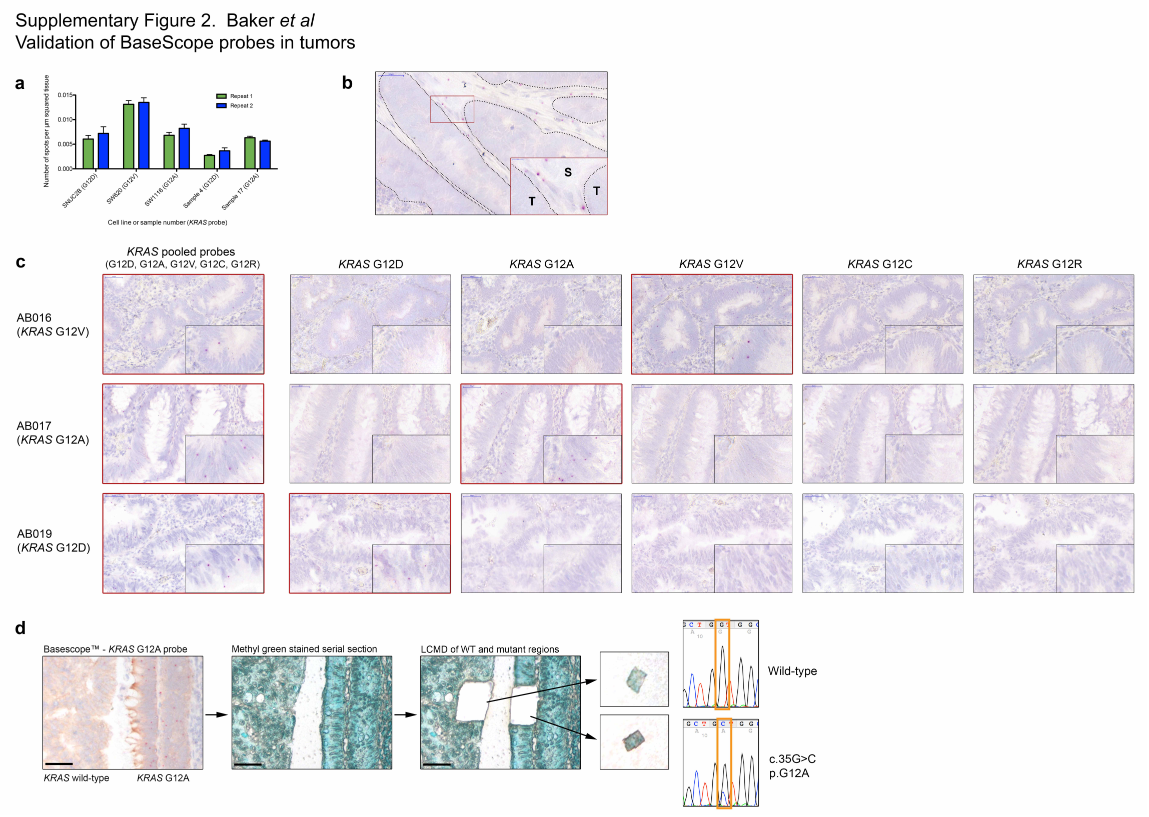

Supplementary Figure 2 – Validation of BaseScope probes in tumors

a. Reproducibility of BaseScope signal quantification. Technical repeats were performed at least 3 months apart, and signal was quantified by counting positive spots in 5 representative fields of view. Bars represent the mean, and error bars represent the standard error of the mean. The difference between means of the two repeats is non-significant (P>0.05) in each case by the Mann-Whitney test.

b. Representative image showing binding of the *KRAS* WT probe only in stromal cells of a *KRAS* G12D homozygous mutant tumor (AB004). S = stroma, T = tumor.

c. Representative images of three cases with *de novo KRAS* mutations detected firstly with the pool of *KRAS* mutant probes (left column), and then tested with each of the 5 individual probes to detect which mutation is present (red box). The probes are entirely specific to the mutation that is present and there is no non-specific signal from the other *KRAS* mutant probes.

Scale bars in panels **b** and **c** represent 50 micron and 10 micron (inset).

d. Validation of the *KRAS* G12A mutation in sample AB017 by laser capture microdissection, DNA extraction and Sanger sequencing of *KRAS*. Scale bars represent 50 micron.

Supplementary Figure 3. Baker *et al*
BaseScope can be combined with IHC or DNA-FISH

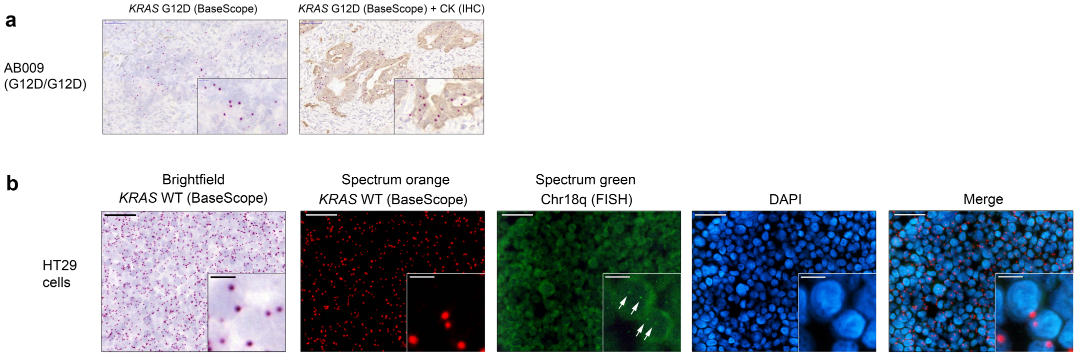

Supplementary Figure 3 – BaseScope can be combined with IHC or DNA-FISH
a. Representative images of *KRAS* G12D BaseScope signal alone (left panel) and with sequential IHC staining for pan-cytokeratin (CK, right panel).
b. Representative images of *KRAS* wild-type BaseScope signal (visible in brightfield and under the 'Spectrum orange' filter) combined with FISH for Chr18q (under the 'Spectrum green' filter, white arrows highlight location of the FISH signals). Scale bars in **a** and **b** represent 50 micron and 10 micron (inset).

Supplementary Figure 4. Baker *et al*
BaseScope detection of CRC subclonal architecture

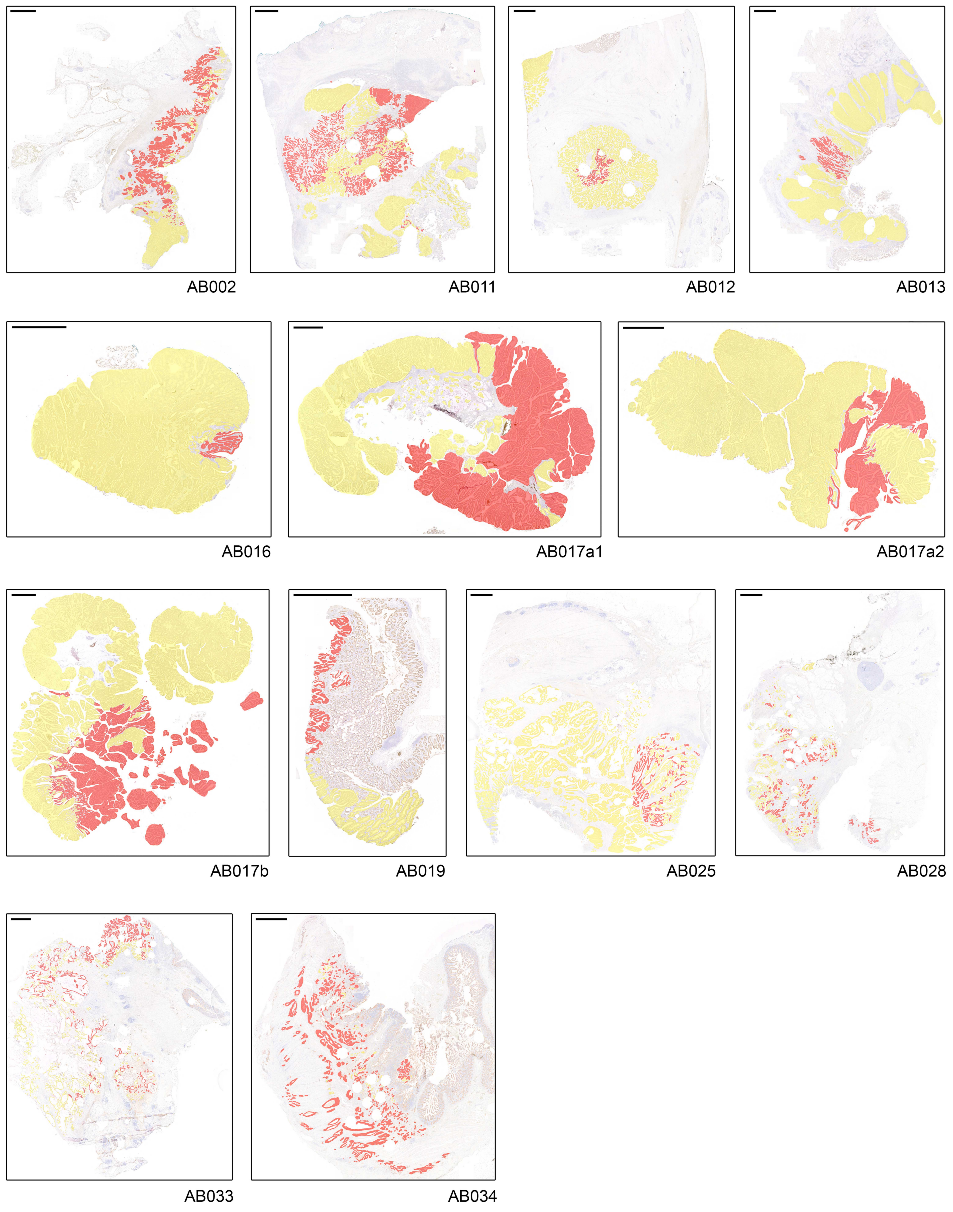

Supplementary Figure 4 – BaseScope detection of CRC subclonal architecture
Topographical maps showing the spatial arrangement of wild-type (yellow) and mutant (red) regions within tumors containing subclonal mutations (n = 13). Scale bars: 2000 microns.

Supplementary Figure 5. Baker *et al*
3D reconstruction shows intimate mixing of KRAS wild-type and mutant subclones.

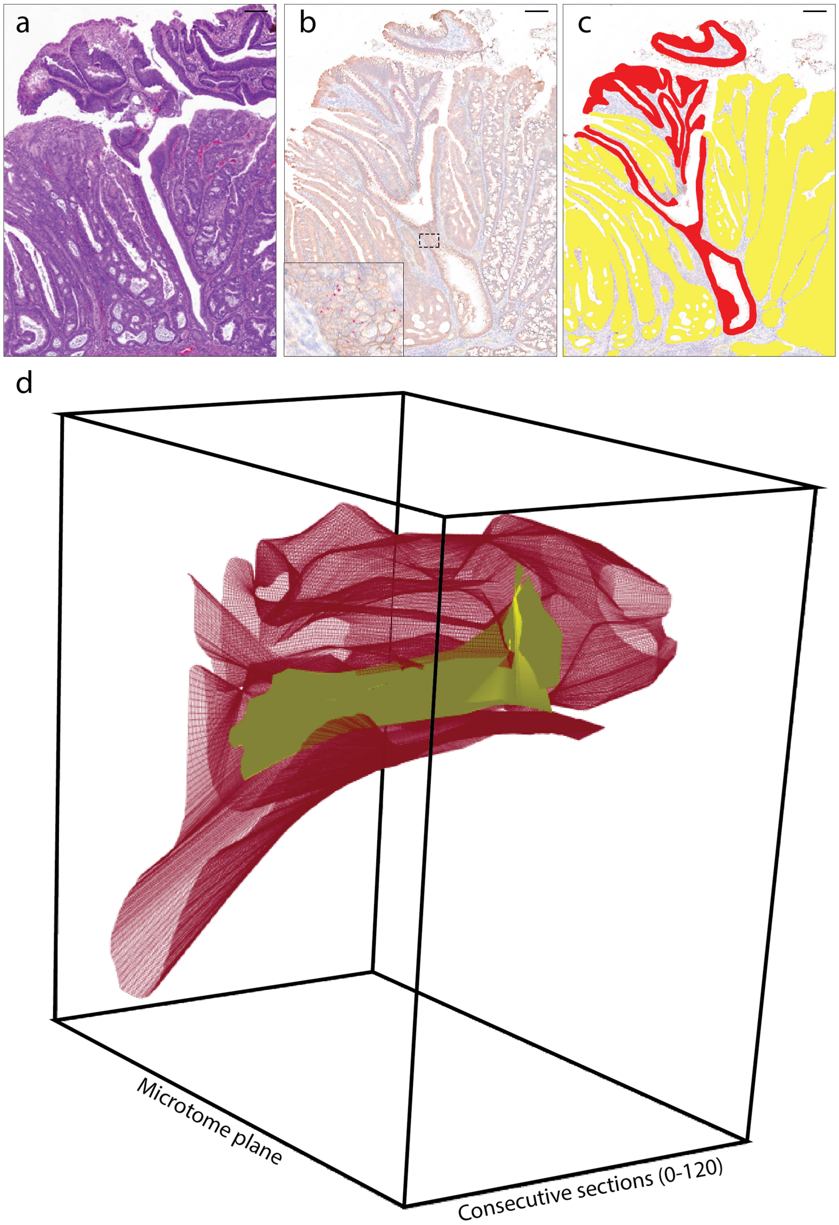

Supplementary Figure 5 - 3D reconstruction shows intimate mixing of *KRAS* wild-type and mutant subclones.

a. Overview H&E shows superficial of a moderately differentiated colorectal adenocarcinoma.

b. Basescope reveals a small *KRAS* mutant subclone.

c. *KRAS* mutant subclone shown in false color (red). The surrounding *KRAS* wild-type is shown in yellow. Note that the *KRAS* mutant subclone appears to enclose *KRAS* wild-type epithelium in the standard 2D section. Scale bars in panels **a-c** represent 200 micron.

d. 3D reconstruction reveals that this wild-type clone is enveloped in *KRAS* mutant epithelium, demonstrating complex spatial mixing behavior.

Supplementary Figure 6. Baker *et al*
Phenotypic analysis of subclones

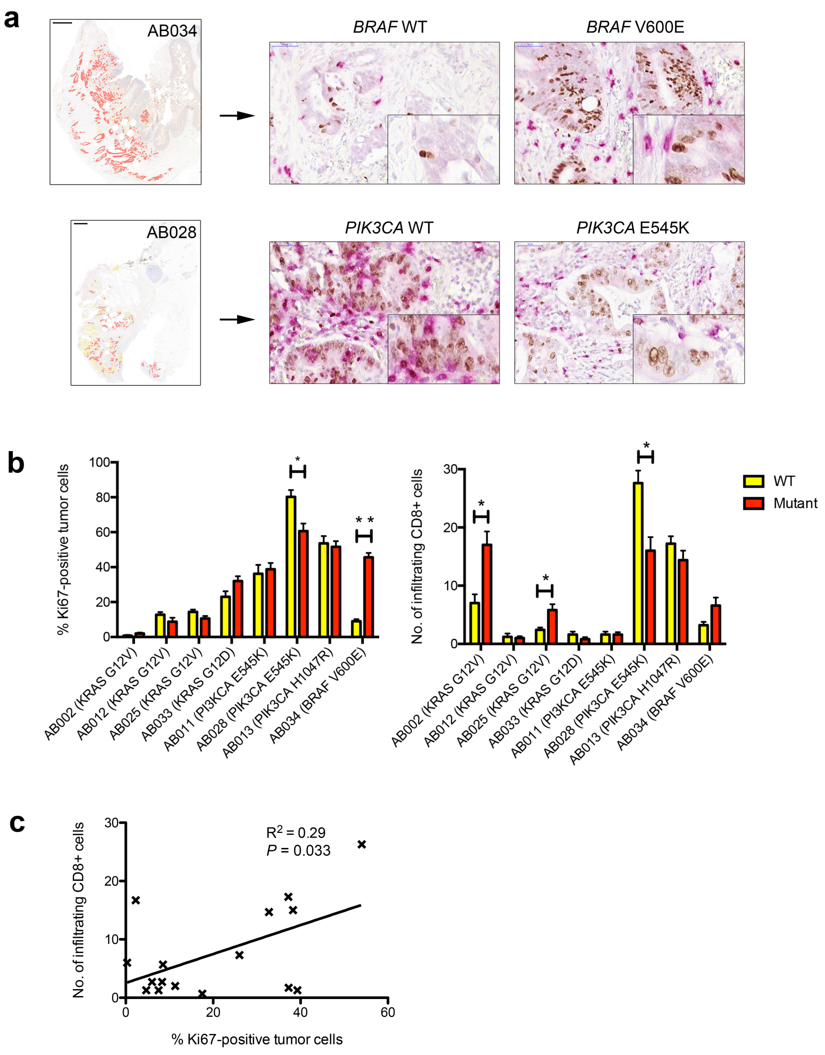

Supplementary Figure 6 – Phenotypic analysis of subclones
a. Representative images showing Ki67 (brown) and CD8 (red) dual-color IHC in two cases. Case AB034 shows higher Ki67 expression and more infiltrating CD8-positive T cells in the *BRAF* V600E mutant subclone. Case AB028 shows lower Ki67 expression and fewer infiltrating CD8-positive T cells in the *PIK3CA* E545K mutant subclone. Scale bars represent 2000 microns (maps), 50 micron and 10 micron (inset).
b. Quantification of Ki67 (left panel) and CD8 (right panel)-positive cells in wild-type and mutant regions of morphologically indistinguishable subclones (**P* < 0.05, ***P* < 0.01 by the Mann-Whitney test). Bars represent the mean, and error bars represent the standard error of the mean. For each of the wild-type and mutant subclones, 5 random areas comprising 100 tumor cells each were counted.
c. Weak correlation between the proportion of Ki67-positive tumor cells and the number of infiltrating CD8-positive cytotoxic T cells in subclonal CRCs (R^2 = 0.29, *P* = 0.033 by the F test).

Supplementary Figure 7. Baker *et al*
Analysis of clone mixing

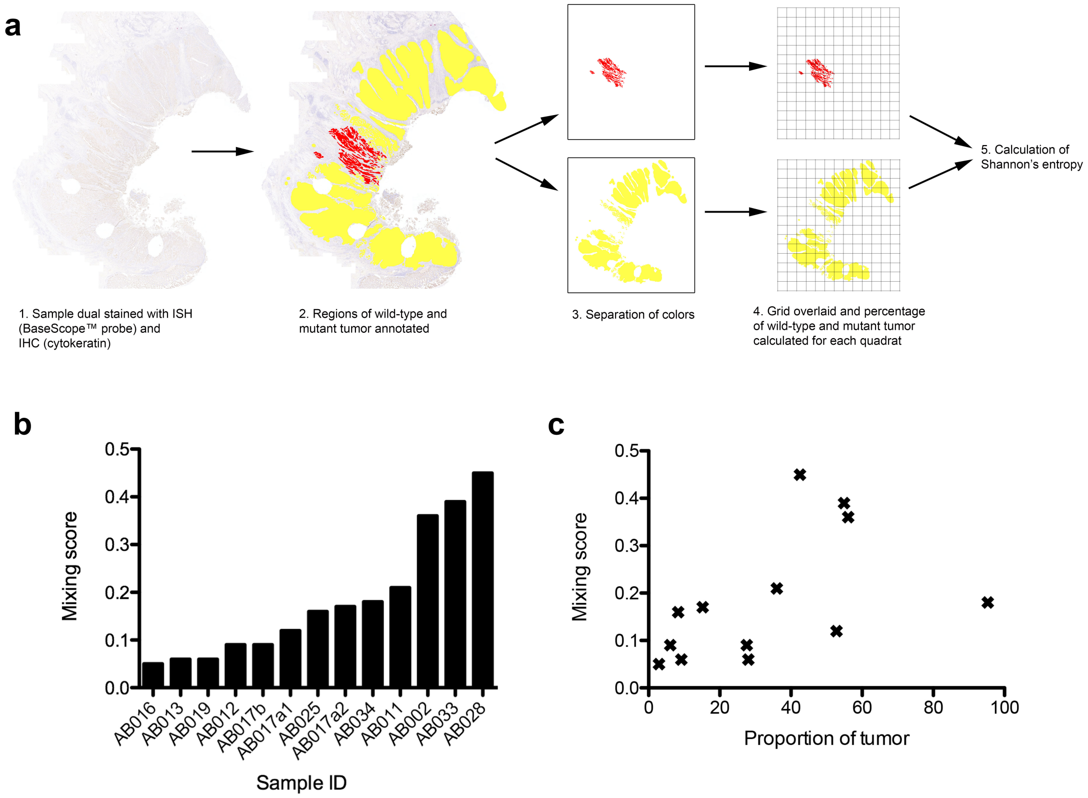

Supplementary Figure 7 – Analysis of clone mixing

a. Schematic showing the methodology of calculating mixing scores (Shannon's entropy) from annotated tissue sections.

b. Mixing scores of each sample.

c. Relationship between the proportion of the lesion occupied by the mutant subclone and the mixing score.

Supplementary Figure 8. Baker *et al*
Spatial modeling predicts the probabilities of observing cloning mixing

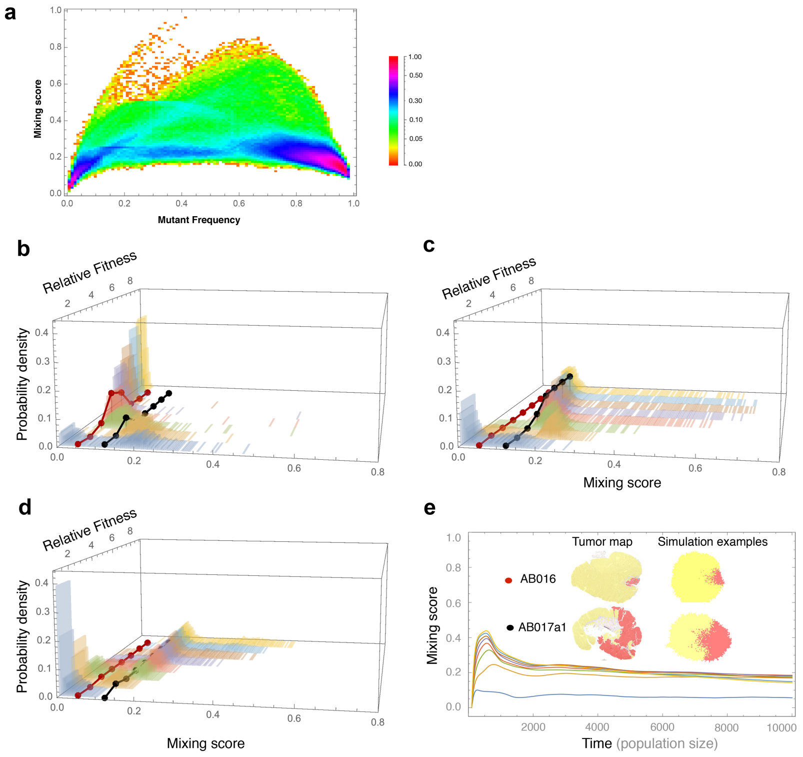

Supplementary Figure 8 – Spatial modeling predicts the degree of clone intermixing

a. Data showing the nonlinear relationship between mutant frequency and mixing score. The mixing scores and mutant frequencies at the end of each simulation were recorded under various selection intensities. The color refers to the density of points in the figure, which has been scaled between 0 and 1. Based on all data points, we see that the mixing score increases when the mutant frequency increases to intermediate values and decreases again when the mutant reaches fixation and thus its frequency approaches to 1. All results are estimated over 1000 independent realizations; the initial mutant frequency is 0.01 and the tumor reaches a final size of 10^4. Simulations assumed that the mutant arises at an intermediate time during tumor expansion.

b-e. Panels **b**, **c** and **d** show the probability density distributions of the spatial mixing (measured by Shannon scoring method with a value between 0 and 1) when the mutant arises (**a**) early, (**b**) intermediate and (**c**) late in tumor expansion. Red and black dots (joined by lines) are examples of probabilities to observe the empirically measured levels of mixing in two samples with relatively low (AB016) and high mixing (AB017a1) respectively. Panel **e** shows how the mixing level changes over time after the mutant appears – the mixing level increases to a transient high at intermediate times before declining as the mutant subclone either takes over the population or is lost. All distributions are results over 1000 independent realizations; in panels **b**, **c**, **d**, initial mutant frequencies are 0.1, 0.01, 0.001 respectively, and the distributions are estimated when the tumor reaches a final size of 10^4.

Supplementary Figure 9. Baker *et al*
Inference of mutant clone fitness and sampling time

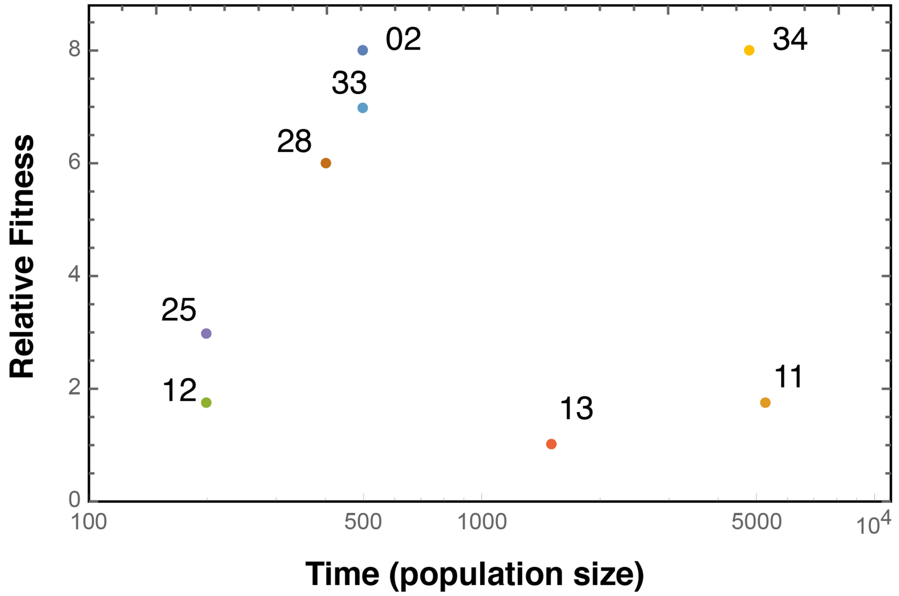

Supplementary Figure 9 – Inference of mutant clone fitness and relative tumor age
Maximum posterior probabilities of subclone selection intensity and age of the CRC (time of surgery relative to tumor initiation) for each case. Samples with low mutant frequency and low subclonal mixing (e.g. sample AB025, AB012, AB013) were best explained if the subclone experienced no or weak selection intensity, whereas samples with intermediate mutant frequency and mixing (e.g. sample AB002, AB028, AB033) were most likely to be observed if the subclone experienced intermediate/high selection intensity and was sampled in an intermediate time after the mutant arose. Only if the selection intensity is strong and the tumor is sampled late after the mutant arose was a high mutant frequency with low mixing (e.g. sample AB034) likely. All results are estimated over 1000 independent realizations; the initial mutant frequency is 0.01 and the tumor reaches a final size of 10^4. Simulations assumed that the mutant arises at an intermediate time during tumor expansion.

Supplementary Figure 10. Baker *et al*
Comparison of 'cell death' and 'pushing' model formulations

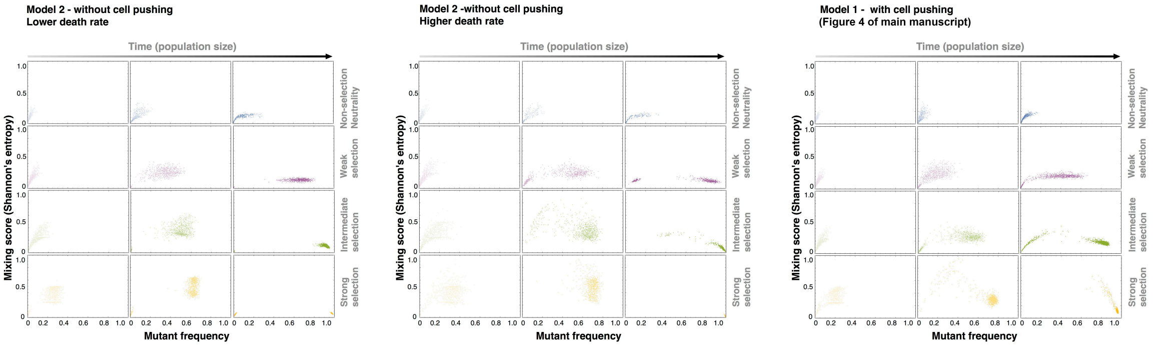

Supplementary Figure 10 – Comparison of 'cell death' and 'pushing' model formulations

The left and middle panels show the results of an alternative model where daughter cells can only divide into neighboring empty spaces, without pushing. In this model, cells die with a fixed rate and so create empty spaces inside the tumor. The right panels show the results of our spatial model with cell pushing during cell division. The qualitative patterns of clone spread and mixing are the same in both models. Under neutral or near-neutral selection (the first row of all panels), mixing and mutant frequency are low irrespective of when the tumor is sampled. When selection intensity is strong, a temporal transit from low mixing to high mixing and then to low mixing again is observed. A high death rate (the middle panel) produces more empty spaces in the population, and mutants with fitness advantages are more likely to reach fixation than in the 'pushing model'. (Each panel shows results from 1000 independent realizations; relative growth ratios of the mutant to wild type are 1, 1.75, 3.0, 8.0 for neutral, weak, intermediate, and strong selection respectively; the initial mutant frequency is 0.01 and the final tumor size is 10^4; death rate is 0.1 and 0.5 for the left and middle panels.)

<u>Supplementary Table 1 – Details of 1ZZ BaseScope probes</u>

Probe Type	Target amino acid variant	Target nucleotide variant	Notes
Negative control – *dapB*	N/A	N/A	
Positive control – *POLR2A*	N/A	N/A	
BRAF V600 WT	N/A	N/A	
BRAF V600 Mutant	p.V600E	c.1799T>A	
KRAS G12 WT (1)	N/A	N/A	Control probe for G12D
KRAS G12 Mutant	p.G12D	c.35G>A	
KRAS G12 WT (2)	N/A	N/A	Control probe for G12A/G12V
KRAS G12 Mutant	p.G12V	c.35G>T	
KRAS G12 Mutant	p.G12A	c.35G>C	
KRAS G12 WT (3)	N/A	N/A	Control probe for G12C/G12R/G12S
KRAS G12 Mutant	p.G12C	c.34G>T	
KRAS G12 Mutant	p.G12R	c.34G>C	
KRAS G12 Mutant	p.G12S	c.34G>A	
PIK3CA E545 WT	N/A	N/A	
PIK3CA E545 Mutant	p.E545K	c.1633G>A	
PIK3CA H1047 WT	N/A	N/A	
PIK3CA H1047 Mutant	p.H1047R	c.3140A>G	

Supplementary Table 2 – Validation of 1ZZ probes in cell lines

Probe set	Cell line	Mutational status at site of interest	*dapB* positive cells (%)	*POLR2A* positive cells (%)	WT probe positive cells (%)	Mutant probe positive cells (%)
BRAF V600E	CHL-1	Wild-type	1/180 (0.6)	214/218 (98.2)	122/209 (58.4)	0/194 (0.0)
	SK-MEL-28	Homozygous mutant	3/174 (1.7)	174/176 (98.9)	1/172 (0.6)	185/193 (95.6)
KRAS G12D	HT29	Wild-type	5/572 (0.9)	581/593 (98.0)	561/590 (95.1)	0/564 (0.0)
	SNU-C2B	Heterozygous mutant	0/288 (0.0)	273/277 (98.6)	192/274 (70.1)	167/294 (56.8)
KRAS G12R	HuT 78	Wild-type	1/474 (0.2)	492/494 (99.6)	315/504 (62.5)	1/511 (0.2)
	PSN-1	Heterozygous mutant	0/344 (0.0)	338/346 (97.7)	297/373 (79.6)	51/312 (16.3)
KRAS G12V	HT29	Wild-type	2/521 (0.4)	502/504 (99.6)	395/458 (86.2)	0/543 (0.0)
	SW620	Homozygous mutant	1/620 (0.2)	573/573 (100.0)	1/635 (0.2)	377/550 (68.5)
KRAS G12A	HuT 78	Wild-type	1/427 (0.2)	401/405 (99.0)	257/419 (61.3)	0/403 (0.0)
	SW1116	Heterozygous mutant	1/499 (0.2)	381/387 (98.4)	223/419 (53.2)	272/427 (63.7)
KRAS G12C	HuT 78	Wild-type	1/409 (0.2)	411/414 (99.3)	318/455 (69.9)	1/426 (0.2)
	SW1573	Homozygous mutant	0/314 (0.0)	354/354 (100.0)	0/376 (0.0)	330/366 (90.2)
KRAS G12S	HuT 78	Wild-type	0/405 (0.0)	425/429 (99.1)	291/423 (68.8)	0/441 (0.0)
	A549	Homozygous mutant	0/220 (0.0)	232/237 (97.9)	0/243 (0.0)	233/265 (87.9)
PIK3CA E545K	BT20	Wild-type	0/367 (0.0)	328/328 (100.0)	366/376 (97.3)	1/342 (0.3)
	MCF-71	Heterozygous mutant	1/389 (0.3)	401/403 (99.5)	243/410 (59.3)	179/322 (55.6)
PIK3CA H1057R	MCF-71	Wild-type	1/347 (0.3)	383/385 (99.5)	393/422 (93.1)	0/377 (0.0)
	BT20	Heterozygous mutant	0/350 (0.0)	319/319 (100.0)	264/302 (87.4)	176/293 (60.1)

Supplementary Table 3 – Details of patient samples

Sample reference	Tumor type	Tumor stage/MSI status	Age of block (years)	Mutational status by sequencing (NGS/Sanger)	Mutational status by BaseScope	Subclonal map
AB001	Colorectal	pT4	2	KRAS WT	KRAS WT*	
AB002a	Colorectal	pT3/MSI+	3	KRAS G12V	KRAS G12V	
AB002b	Colorectal	pT3/MSI+	3	KRAS G12V	KRAS G12V	Yes
AB003	Colorectal	pT4	2	KRAS G13D, BRAF WT	BRAF WT	
AB004	Colorectal	pT4	2	KRAS G12D, BRAF WT	KRAS G12D, BRAF WT	
AB005	Colorectal	pT2	3	KRAS G12D, BRAF WT	KRAS G12D, BRAF WT	
AB006	Colorectal	pT4	2	BRAF V600E	BRAF V600E	
AB007	Colorectal	pT4	2	BRAF V600E	BRAF V600E	
AB008	Colorectal	pT4	0	BRAF V600E	Unknown	
AB009	Colorectal	Unknown	8	KRAS G12D	KRAS G12D	
AB010	Colorectal	Unknown	11	KRAS G12C	KRAS G12C	
AB011	Colorectal	Unknown	7	PIK3CA E545K	PIK3CA E545K	Yes
AB012	Colorectal	Unknown	10	KRAS G12V	KRAS G12V	Yes
AB013	Colorectal	MSI+	9	PIK3CA H1047R	PIK3CA H1047R	Yes
AB014	Colorectal	Unknown	9	PIK3CA E545K	PIK3CA E545K	
AB015	Colorectal	pT1	8	BRAF V600E	BRAF V600E	
AB016	Colorectal	pT1 (Ca-in-ad)	3	KRAS WT	KRAS G12V	Yes
AB017a	Colorectal	pT1 (Ca-in-ad)	3	KRAS WT	KRAS G12A	Yes (x2)
AB017b	Colorectal	pT1 (Ca-in-ad)	3	KRAS WT	KRAS G12A	Yes
AB017c	Colorectal	pT1 (Ca-in-ad)	3	KRAS WT	KRAS WT*	
AB018	Colorectal	pT1 (Ca-in-ad)	2	KRAS WT	KRAS WT*	
AB019a	Colorectal	pT1 (Ca-in-ad)	4	KRAS WT	KRAS G12D	Yes
AB019b	Colorectal	pT1 (Ca-in-ad)	4	KRAS WT	KRAS WT*	
AB019c	Colorectal	pT1 (Ca-in-ad)	4	KRAS WT	KRAS WT*	
AB020	Colorectal	pT1 (Ca-in-ad)	2	KRAS WT	KRAS WT*	
AB021	Colorectal	pT1 (Ca-in-ad)	2	KRAS WT	KRAS WT*	
AB022	Colorectal	pT1 (Ca-in-ad)	2	KRAS WT	KRAS WT*	
AB023	Colorectal	pT1 (Ca-in-ad)	2	KRAS WT	KRAS WT*	
AB024	Colorectal	pT1 (Ca-in-ad)	3	KRAS WT	KRAS WT*	
AB025	Colorectal	Unknown	10	KRAS G12V	KRAS G12V	Yes
AB026	Colorectal	Unknown	9	KRAS G12V	KRAS G12V	
AB027	Colorectal	Unknown	15	PIK3CA E545K	PIK3CA E545K	
AB028	Colorectal	Unknown	14	PIK3CA E545K	PIK3CA E545K	Yes
AB029	Colorectal	Unknown	20	KRAS G12D	KRAS G12D	
AB030	Colorectal	Unknown	14	BRAF V600E	BRAF V600E	
AB031	Colorectal	Unknown	14	PIK3CA E545K	PIK3CA E545K	
AB032	Colorectal	Unknown	13	KRAS G12C	KRAS G12C	
AB033	Colorectal	Unknown	15	KRAS G12D	KRAS G12D	Yes
AB034	Colorectal	Unknown	16	BRAF V600E	BRAF V600E	Yes
AB035	Colorectal	Unknown	15	KRAS G12C	KRAS G12C	
AB036	Colorectal	Unknown	14	BRAF WT	BRAF WT	
AB037	Colorectal	Unknown	16	KRAS WT	KRAS WT*	
AB038	Melanoma	Unknown	0	BRAF V600E	BRAF V600E	
AB039	Colorectal	pT1 (Ca-in-ad)	2	KRAS WT	KRAS WT*	
AB040	Colorectal	pT1 (Ca-in-ad)	2	KRAS WT	KRAS WT*	
AB041	Colorectal	pT1 (Ca-in-ad)	3	KRAS WT	KRAS WT*	
AB042	Colorectal	pT1 (Ca-in-ad)	2	KRAS WT	KRAS WT*	
AB043	Colorectal	pT1 (Ca-in-ad)	4	KRAS WT	KRAS WT*	
AB044	Colorectal	pT1 (Ca-in-ad)	4	KRAS WT	KRAS WT*	
AB045	Colorectal	pT1 (Ca-in-ad)	4	KRAS WT	KRAS WT*	
AB046	Colorectal	pT1 (Ca-in-ad)	2	KRAS WT	KRAS WT*	

KRAS WT* = a mutation was not detected using the probes G12D, G12V, G12A, G12C, G12R, G12S; MSI = microsatellite instable.

Supplementary Table 4 – Validation of 1ZZ probes in human tumors

Sample reference	Tumor type	Mutational status at site of interest	*dapB* positive cells (%)	*POLR2A* positive cells (%)	*KRAS* WT positive cells (%)	*KRAS* G12D positive cells (%)	*BRAF* WT positive cells (%)	*BRAF* V600E positive cells (%)
AB001	CRC	KRAS WT<br>BRAF WT	0/250<br>(0.0)	127/251<br>(50.6)	143/251<br>(57.0)	1/236<br>(0.4)	ND	ND
AB003	CRC	KRAS G13D<br>BRAF WT	1/197<br>(0.5)	83/217<br>(38.2)	14/202<br>(6.9)	0/228<br>(0.0)	ND	ND
AB004	CRC	KRAS G12D<br>BRAF WT	0/312<br>(0.0)	262/294<br>(89.1)	0/286<br>(0.0)	44/265<br>(16.6)	139/305<br>(45.6)	0/308<br>(0.0)
AB005	CRC	KRAS G12D<br>BRAF WT	ND	ND	50/316<br>(15.8)	125/279<br>(44.8)	ND	ND
AB007	CRC	KRAS unknown<br>BRAF V600E	0/174<br>(0.0)	227/264<br>(86.0)	ND	ND	25/220<br>(11.4)	29/222<br>(13.1)
AB038	Melanoma	KRAS unknown<br>BRAF V600E	1/269<br>(0.4)	ND	ND	ND	68/269<br>(25.3)	56/293<br>(19.1)

An average of 238 (+/- 30) tumor cells over three fields of view were counted for each probe in each tumor. ND = not done.

Supplementary Table 5 – Details of subclonal maps

Sample	Mutation	Percentage of tumor mutant	Shannon's entropy	Sample Type
AB002	KRAS G12V	56.1	0.36	CRC
AB011	PIK3CA E545K	36.0	0.21	CRC
AB012	KRAS G12V	6.1	0.09	CRC
AB013	PIK3CA H1047R	9.2	0.06	CRC
AB016	KRAS G12V	2.9	0.05	Ca-in-ad
AB017a1	KRAS G12A	52.8	0.12	Ca-in-ad
AB017a2	KRAS G12A	15.2	0.17	Ca-in-ad
AB017b	KRAS G12A	27.6	0.09	Ca-in-ad
AB019	KRAS G12D	28.0	0.06	Ca-in-ad
AB025	KRAS G12V	8.3	0.16	CRC
AB028	PIK3CA E545K	42.5	0.45	CRC
AB033	KRAS G12D	54.9	0.39	CRC
AB034	BRAF V600E	95.3	0.18	CRC
