# Supplementary material for: Robust RNA-based in situ mutation detection delineates colorectal cancer subclonal evolution
Source: Nat Commun. 2017 Dec 8;8:1998. doi: 10.1038/s41467-017-02295-5 (PMC5722928; doi:10.1038/s41467-017-02295-5)
Supplement: Supplementary file 3 — Description of Additional Supplementary Files [file 41467_2017_2295_MOESM3_ESM.pdf]

Description of Supplementary Files

File Name: Supplementary Movie 1
Description: **3D reconstruction of clone mixing**. Movie showing the complex and intimate mixing of a mutant clone (red) and a wild type clone (yellow), reconstructed from serial BaseScope-stained sections.
